# Supplementary material for: Physical harms in colorectal cancer screening: An overview of the reporting in systematic reviews and randomised controlled trials
Source: PLoS One. 2025 Sep 11;20(9):e0331104. doi: 10.1371/journal.pone.0331104 (PMC12425235; doi:10.1371/journal.pone.0331104)
Supplement: S1 File — S1 includes Appendices A–I, which contain information and data that support the manuscript. (DOCX) [file pone.0331104.s001.docx]

# **S1 Appendices A to I**

# **Appendix A: The 17 types of physical harm**

This appendix presents the 17 identified types of physical harm identified in the reference review, and used in the analysis of the current study.

Death

Perforation

Cardiovascular- and pulmonary complications

Bleeding

Post-polypectomy syndrome

Infections

Inflammatory complications

Colorectal symptoms

Sedation-related complications

Complications related to bowel preparation

Sleep disturbances

Nausea/vomiting

Dizziness

Pain

Discomfort

Other harms

Complications/adverse events in total

# **Appendix B: Search strategy**

This appendix details the full search strategy applied in the systematic literature search, including search terms, databases searched, date of last search, and any applied filters or limits.

Overall search principle:

((Screening AND laxative) OR (screening AND CRC) OR CRCSPs) AND physical harm, limit: systematic reviews

**Pubmed search 10-07-2024:**

Search: (((("Colonoscopy"[Mesh] OR "Sigmoidoscopy"[Mesh] OR "Occult Blood"[Mesh] OR "Colonic Neoplasms/surgery"[Mesh] OR "Intestinal Polyps/surgery"[Mesh] OR "Colorectal Neoplasms/prevention and control"[Mesh])) OR (((("intestinal polyps"[MeSH Terms] OR "cecal neoplasms"[MeSH Terms] OR "colorectal neoplasms"[MeSH Terms]) OR ("adenoma"[MeSH Terms] AND ("Intestine, Large"[Mesh] OR "Intestinal Neoplasms"[Mesh])))) AND ("Early Diagnosis"[Mesh] OR "Secondary Prevention"[Mesh] OR "Early Detection of Cancer"[Mesh] OR "Mass Screening"[Mesh] OR "Preventive Health Services"[Mesh] OR "Early Medical Intervention"[Mesh]))) OR ((((("Colonoscopy"[Mesh] OR "Sigmoidoscopy"[Mesh] OR "Occult Blood"[Mesh] OR "Colonic Neoplasms/surgery"[Mesh] OR "Intestinal Polyps/surgery"[Mesh] OR "Colorectal Neoplasms/prevention and control"[Mesh])) OR ("Early Diagnosis"[Mesh] OR "Secondary Prevention"[Mesh] OR "Early Detection of Cancer"[Mesh] OR "Mass Screening"[Mesh] OR "Preventive Health Services"[Mesh] OR "Early Medical Intervention"[Mesh]))) AND ("Cathartics"[Mesh] OR "Laxatives"[Mesh]))) AND ("Postoperative Complications"[Mesh] OR "Intraoperative Complications"[Mesh] OR "Death"[Mesh] OR "Pain"[Mesh] OR "Pain Measurement"[Mesh] OR "Signs and Symptoms, Digestive"[Mesh] OR "Wound Infection"[Mesh] OR "Blood Loss, Surgical"[Mesh] OR "Heart Diseases"[Mesh] OR "Hypovolemia"[Mesh] OR "Ischemia"[Mesh] OR "Hypersensitivity"[Mesh] OR "Inflammation"[Mesh] OR "Shock"[Mesh] OR "Ulcer"[Mesh] OR "Intestinal Perforation"[Mesh] OR "Abdominal Injuries"[Mesh] OR "Iatrogenic Disease"[Mesh] OR "Medical Errors"[Mesh] OR "Safety"[Mesh] OR "Long Term Adverse Effects"[Mesh] OR "Water-Electrolyte Imbalance"[Mesh] OR "Colon/injuries"[Mesh] OR "Early Diagnosis/adverse effects"[Mesh] OR "Early Diagnosis/mortality"[Mesh] OR "Early Detection of Cancer/adverse effects"[Mesh] OR "Early Detection of Cancer/mortality"[Mesh] OR "Mass Screening/adverse effects"[Mesh] OR "Mass Screening/mortality"[Mesh] OR "Preventive Health Services/adverse effects"[Mesh] OR "Preventive Health Services/complications"[Mesh] OR "Preventive Health Services/mortality"[Mesh] OR "Colonoscopy/adverse effects"[Mesh] OR "Colonoscopy/complications"[Mesh] OR "Colonoscopy/mortality"[Mesh] OR "Sigmoidoscopy/adverse effects"[Mesh] OR "Sigmoidoscopy/complications"[Mesh] OR "Sigmoidoscopy/mortality"[Mesh] OR "Occult Blood/adverse effects"[Mesh] OR "Cathartics/adverse effects"[Mesh] OR "Cathartics/poisoning"[Mesh] OR "Cathartics/toxicity"[Mesh] OR "Laxatives/adverse effects"[Mesh] OR "Laxatives/poisoning"[Mesh] OR "Laxatives/toxicity"[Mesh]) Filters: Systematic Review

# **Appendix C Eligibility criteria** This appendix outlines the predefined inclusion and exclusion criteria used to assess the eligibility of studies for the overview.

|  | Inclusion | Exclusion |
| --- | --- | --- |
| Population | 40 years < Age < 80 years*  Mean risk of CRC and asymptomatic regarding signs of CRC (screening population)^1^. | People at higher-than-average risk of CRC. People with family history of CRC, known genetic susceptibility to CRC or known IBD. |
|  |  | Non-screening populations (symptomatic, earlier or current diagnosis of CRC) |
| Setting | Settings corresponding to clinical screening settings in real life | Research or industrial based settings that diverge from normal screening setting to an extent that comparison with screening setting is not possible. Including general practice or specialized endoscopic treatment centers. |
| Intervention | Conventional CRC screening tools, defined as all the following screening techniques in any type of combination: Faecal occult blood test (guaiac or immunochemical tests), sigmoidoscopy and colonoscopy. | Stool testing using in-office digital rectal exam (DRE). Genetic testing for increased risk of CRC. Blood tests such as septins. Stool DNA test. Capsule endoscopy or CT colonography.  Any interventions after the screening colonoscopy/sigmoidoscopy, i.e. people referred for further work-up/treatment or people participating in surveillance programmes due to former identification of cancer or cancer precursors, i.e. polyps. Any interventions other than conventional screening methods designed to reduce harm, e.g. music therapy, pain medication etc. |
| Outcomes | Any type of physical harm no matter its severity, potential causality or consequences, thus comprising the totality of adverse consequences deemed physical in nature. Physical harms occurring due to the diagnostic work-up procedures of CRCSPs: sigmoidoscopy and/or colonoscopy with or without polypectomy. No restrictions on follow-up time. | Expected harms of screening from participants or harm of screening in the view of physicians or other health professionals. Including simulated numbers of harm in microsimulation studies. Any study not reporting about harms in title, abstract or full text. |
| Study design | RCTs and systematic reviews | Any article not related to original data/research: Journalism, editorials, narrative reviews, and opinions as letters or comments.  All other study designs than RCTs and systematic reviews, e.g. qualitative studies, observational studies etc.  We excluded smaller studies, e.g. feasibility studies, and studies that were only reported in abstract format because we could not expect the same reporting quality of physical harms as in full-text publications from these.  Studies reporting harm while comparing technical aspects of procedures such as type of colonoscope, different anaesthetic procedures, different bowel preparation schemes or different polypectomy techniques. |
| ^1^Mixed populations were included if data was stratified corresponding to these eligibility criteria or when most of the study population fitted the eligibility criteria, i.e. small discrepancies are accepted. ^2^Reference lists in reviews deemed relevant to the research question will be scrutinized for studies not found via the search strategy. *We accepted studies that included people above and below the desired age interval as many screening programmes provide the opportunity for people outside the age group to opt-in.  Abbreviations: CRC = Colorectal cancer, IBD = Inflammatory Bowel Disease | | |

# **Appendix D: Data extraction strategy**

This appendix describes the procedure for data extraction.

**Data extracted from RCTs:**

1. **Study ID (First author - year)**
2. **Referring to study protocol?**
   1. **YES/NO**
   2. **Important deviations from protocol?**
      1. **Free text**
3. **Year of publication**
4. **Country**
5. **Study period**
6. **People screened**
7. **Sex distribution, % women**
8. **Age interval of study population**
9. **Types of harm reported – see appendix E for categorization**

**Data extracted from SRs:**

1. **Study ID (First author - year)**
2. **Referring to study protocol?**
   1. **YES/NO**
   2. **Important deviations from protocol?**
      1. **Free text**
3. **Year of publication**
4. **Data sources**
5. **Time period included**
6. **Study designs included for review**
7. **Language restrictions**
8. **Number of studies included for review**
9. **Population characteristics**
10. **Types of harm reported – see appendix E for categorization**

# **Appendix E: Rules for categorisation and severity assessment**

This appendix explains the rules and criteria used to categorise types of harm and to assess their severity.

It was decided, that for a specific outcome to qualify as a “harm type”, the outcome had to be reported in at least three different studies^[[1]](#footnote-1)^.

When categorising outcomes in “types of harms”, the following rules applied:

- Symptoms like diarrhea, dizziness, and nausea were always assessed as being Not Severe unless otherwise specified.
- Discomfort was always assessed as being Not Severe, even when specified as being very uncomfortable, etc.
- Pain is considered Not Severe unless otherwise specified - i.e., the outcome “severe pain” will be classified as being Severe, whereas “pain” will be classified as Not Severe. Pain leading to an incomplete procedure will also be classified as being Severe.
- Infections like pneumonia can be very dangerous or almost insignificant depending on age, comorbidity, etc. For this reason, it was categorically assessed as being Unknown unless otherwise specified.
- Incontinence was assessed as Unknown as none of the studies elaborated on the timeframe of the complication. Incontinence lasting one day is not very severe, whereas incontinence for a long period can be quite disabling.
- Perforation of the bowel was always considered Severe unless otherwise specified. The same was presumed for major bleeding and major complications.
- Complications that had a high risk of leading to mortality or serious morbidity were considered Very Severe – e.g. myocardial infarction.
- A complication leading to hospitalization was considered Severe, whereas a complication leading to surgery was considered Very Severe.
- The outcome “Complications/adverse events in total” was only registered as such if they summed up individual categories or explicitly wrote which types of harm were included in the term.

# **Appendix F(a): RCTs identified and assessed at study level (included and excluded)**

| **No.** | **Author** | **Journal** | **Year** | **Title** | **Note** |
| --- | --- | --- | --- | --- | --- |
| 1 | Atkin, W. S.  Hart, A.  Edwards, R.  McIntyre, P.  Aubrey, R.  Wardle, J.  Sutton, S.  Cuzick, J.  Northover, J. M. A. | Gut | 1998 | Uptake, yield of neoplasia, and adverse effects of flexible sigmoidoscopy screening | Excluded: feasibility study |
| 2 | Atkin, W. S.  Cook, C. F.  Cuzick, J.  Edwards, R.  Northover, J. M.  Wardle, J. | The Lancet | 2002 | Single flexible sigmoidoscopy screening to prevent colorectal cancer: baseline findings of a UK multicentre randomised trial | Included |
| 3 | Blom, J.  Lidén, A.  Nilsson, J.  Påhlman, L.  Nyrén, O.  Holmberg, L. | European Journal of Surgical Oncology | 2004 | Colorectal cancer screening with flexible sigmoidoscopy—participants’ experiences and technical feasibility | Excluded: feasibility study |
| 4 | Bretthauer, M.  Kaminski, M. F.  Loberg, M.  Zauber, A. G.  Regula, J.  Kuipers, E. J.  Hernan, M. A.  McFadden, E.  Sunde, A.  Kalager, M.  Dekker, E.  Lansdorp-Vogelaar, I.  Garborg, K.  Rupinski, M.  Spaander, M. C.  Bugajski, M.  Hoie, O.  Stefansson, T.  Hoff, G.  Adami, H. O. | **Journal of the American Medical Association (JAMA)** Internal Medicine | 2016 | Population-Based Colonoscopy Screening for Colorectal Cancer: A Randomized Clinical Trial | Included |
| 5 | de Wijkerslooth, T. R.  de Haan, M. C.  Stoop, E. M.  Bossuyt, P. M.  Thomeer, M.  Essink-Bot, M. L.  van Leerdam, M. E.  Fockens, P.  Kuipers, E. J.  Stoker, J.  Dekker, E. | Gut | 2012 | Burden of colonoscopy compared to non-cathartic CT-colonography in a colorectal cancer screening programme: randomised controlled trial | Included |
| 6 | Forbes, G. M.  Mendelson, R. M.  Edwards, J. T.  Foster, N. M.  Bampton, P. A.  Voyvodic, F.  Upton, J.  Macrae, F. A.  Stella, D.  Viney, B.  Pizzey, C. J.  Fritschi, L.  Heyworth, J. | Medical Journal of Australia | 2006 | A comparison of colorectal neoplasia screening tests: a multicentre community-based study of the impact of consumer choice | Included |
| 7 | Fritzell, A.  Forsberg, A.  Wangmar, J.  Wengström, Y.  Bottai, M.  Hultcrantz, R. | Scandinavian Journal of Gastroenterology | 2020 | Gender, having a positive FIT and type of hospital are important factors for colonoscopy experience in colorectal cancer screening – findings from the SCREESCO study | Included |
| 8 | Gondal, G.  Grotmol, T.  Hofstad, B.  Bretthauer, M.  Eide, T. J.  Hoff, G. | Scandinavian Journal of Gastroenterology | 2003 | The Norwegian Colorectal Cancer Prevention (NORCCAP) screening study: baseline findings and implementations for clinical work-up in age groups 50-64 years | Included |
| 9 | Hoff, G.  Grotmol, T.  Skovlund, E.  Bretthauer, M. | British Medical Journal | 2009 | Risk of colorectal cancer seven years after flexible sigmoidoscopy screening: randomised controlled trial | Included |
| 10 | Hol, L.  van Leerdam, M. E.  van Ballegooijen, M.  van Vuuren, A. J.  van Dekken, H.  Reijerink, J. C.  van der Togt, A. C.  Habbema, J. D.  Kuipers, E. J. | Gut | 2010 | Screening for colorectal cancer: randomised trial comparing guaiac-based and immunochemical faecal occult blood testing and flexible sigmoidoscopy | Included |
| 11 | Hol, L.  de Jonge, V.  van Leerdam, M. E.  van Ballegooijen, M.  Looman, C. W.  van Vuuren, A. J.  Reijerink, J. C.  Habbema, J. D.  Essink-Bot, M. L.  Kuipers, E. J. | European Journal of Cancer | 2010 | Screening for colorectal cancer: comparison of perceived test burden of guaiac-based faecal occult blood test, faecal immunochemical test and flexible sigmoidoscopy | Included |
| 12 | Holme, Ø.  Løberg, M.  Kalager, M.  Bretthauer, M.  Hernán, M. A.  Aas, E.  Eide, T. J.  Skovlund, E.  Schneede, J.  Tveit, K. M.  Hoff, G. | **Journal of the American Medical Association (JAMA).** | 2014 | Effect of Flexible Sigmoidoscopy Screening  on Colorectal Cancer Incidence and Mortality  A Randomized Clinical Trial | Included |
| 13 | Kewenter, J.  Brevinge, H. | Diseases of the Colon and Rectum | 1996 | Endoscopic and surgical complications of work-up in screening for colorectal cancer | Included |
| 14 | Kobiela, J.  Spychalski, P.  Wieszczy, P.  Pisera, M.  Pilonis, N.  Rupinski, M.  Bugajski, M.  Regula, J.  Kaminski, M. F. | Clinical Gastroenterology and Hepatology | 2020 | Mortality and Rate of Hospitalization in a Colonoscopy Screening Program From a randomized Health Services Study | Included |
| 15 | Larsen, I. K.  Grotmol, T.  Bretthauer, M.  Gondal, G.  Huppertz-Hauss, G.  Hofstad, B.  Efskind, P.  Jorgensen, A.  Hoff, G. | Scandinavian Journal of Gastroenterology | 2002 | Continuous evaluation of patient satisfaction in endoscopy centres | Included |
| 16 | Mandel, J. S.  Bond, J. H.  Church, T. R.  Snover, D. C.  Bradley, G. M.  Schuman, L. M.  Ederer, F. | New England Journal of Medicine | 1993 | Reducing mortality from colorectal cancer by screening for fecal occult blood. Minnesota Colon Cancer Control Study | Included |
| 17 | Quintero, E.  Castells, A.  Bujanda, L.  Cubiella, J.  Salas, D.  Lanas, A.  Andreu, M.  Carballo, F.  Morillas, J. D.  Hernandez, C.  Jover, R.  Montalvo, I.  Arenas, J.  Laredo, E.  Hernandez, V.  Iglesias, F.  Cid, E.  Zubizarreta, R.  Sala, T.  Ponce, M.  Andres, M.  Teruel, G.  Peris, A.  Roncales, M. P.  Polo-Tomas, M.  Bessa, X.  Ferrer-Armengou, O.  Grau, J.  Serradesanferm, A.  Ono, A.  Cruzado, J.  Perez-Riquelme, F.  Alonso-Abreu, I.  de la Vega-Prieto, M.  Reyes-Melian, J. M.  Cacho, G.  Diaz-Tasende, J.  Herreros-de-Tejada, A.  Poves, C.  Santander, C.  Gonzalez-Navarro, A | New England Journal of Medicine | 2012 | Colonoscopy versus fecal immunochemical testing in colorectal-cancer screening | Included |
| 18 | Randel, K. R.  Schult, A. L.  Botteri, E.  Hoff, G.  Bretthauer, M.  Ursin, G.  Natvig, E.  Berstad, P.  Jørgensen, A.  Sandvei, P. K.  Olsen, M. E.  Frigstad, S. O.  Darre-Næss, O.  Norvard, E. R.  Bolstad, N.  Kørner, H.  Wibe, A.  Wensaas, K.  De Lange, T.  Holme, Ø. | Gastroenterology | 2021 | Colorectal Cancer Screening With Repeated Fecal Immunochemical Test Versus sigmoidoscopy: Baseline Results From a Randomized Trial | Included |
| 19 | Rasmussen, M.  Kronborg, O.  Fenger, C.  Jorgensen, O. D. | Scandinavian Journal of Gastroenterology | 1999 | Possible advantages and drawbacks of adding flexible sigmoidoscopy to hemoccult-II in screening for colorectal cancer. A randomized study | Included |
| 20 | Robertson, D. J.  Dominitz, J. A.  Beed, A.  Ahnen, D.  Boardman, K. D.  Del Curto, B.  Guarino, P.  Imperiale, T. F.  Johnson, G. R.  Kyriakides, T.  Larson, M.  LaCasse, A. J.  Lieberman, D. A.  Provenzale, D.  Shaukat, A.  Sultan, S.  Planeta, B. M. | Gastroenterology | 2017 | **Complications of Screening Colonoscopy in a Multi-Center Study of Colorectal Cancer Screening** | Excluded: abstract only |
| 21 | Robinson, M. H.  Hardcastle, J. D.  Moss, S. M.  Amar, S. S.  Chamberlain, J. O.  Armitage, N. C.  Scholefield, J. H.  Mangham, C. M. | Gut | 1999 | The risks of screening: data from the Nottingham randomised controlled trial of faecal occult blood screening for colorectal cancer | Included |
| 22 | Schoen, R. E.  Pinsky, P. F.  Weissfeld, J. L.  Yokochi, L. A.  Church, T.  Laiyemo, A. O.  Bresalier, R.  Andriole, G. L.  Buys, S. S.  Crawford, E. D.  Fouad, M. N.  Isaacs, C.  Johnson, C. C.  Reding, D. J.  O'Brien, B.  Carrick, D. M.  Wright, P.  Riley, T. L.  Purdue, M. P.  Izmirlian, G.  Kramer, B. S.  Miller, A. B.  Gohagan, J. K.  Prorok, P. C.  Berg, C. D. | New England Journal of Medicine | 2012 | Colorectal cancer incidence and mortality with screening flexible sigmoidoscopy | Included |
| 23 | Segnan, N.  Senore, C.  Andreoni, B.  Aste, H.  Bonelli, L.  Crosta, C.  Ferraris, R.  Gasperoni, S.  Penna, A.  Risio, M.  Rossini, F. P.  Sciallero, S.  Zappa, M.  Atkin, W. S. | Journal of the National Cancer Institute | 2002 | Baseline findings of the Italian multicenter randomized controlled trial of "once-only sigmoidoscopy"--SCORE | Included |
| 24 | Segnan, N.  Senore, C.  Andreoni, B.  Arrigoni, A.  Bisanti, L.  Cardelli, A.  Castiglione, G.  Crosta, C.  DiPlacido, R.  Ferrari, A.  Ferraris, R.  Ferrero, F.  Fracchia, M.  Gasperoni, S.  Malfitana, G.  Recchia, S.  Risio, M.  Rizzetto, M.  Saracco, G.  Spandre, M.  Turco, D.  Turco, P.  Zappa, M. | Journal of the National Cancer Institute | 2005 | Randomized trial of different screening strategies for colorectal cancer: patient response and detection rates | Included |
| 25 | Senore, C.  Ederle, A.  Fantin, A.  Andreoni, B.  Bisanti, L.  Grazzini, G.  Zappa, M.  Ferrero, F.  Marutti, A.  Giuliani, O.  Armaroli, P.  Segnan, N. | Journal of Medical Screening | 2011 | Acceptability and side-effects of colonoscopy and sigmoidoscopy in a screening setting | Included |
| 26 | Stoop, E. M.  de Haan, M. C.  de Wijkerslooth, T. R.  Bossuyt, P. M.  van Ballegooijen, M.  Nio, C. Y.  van de Vijver, M. J.  Biermann, K.  Thomeer, M.  van Leerdam, M. E.  Fockens, P.  Stoker, J.  Kuipers, E. J.  Dekker, E. | The Lancet Oncology | 2012 | Participation and yield of colonoscopy versus non-cathartic CT colonography in population-based screening for colorectal cancer: a randomised controlled trial | Included |
| 27 | van Dam, L.  de Wijkerslooth, T. R.  de Haan, M. C.  Stoop, E. M.  Bossuyt, P. M.  Fockens, P.  Thomeer, M.  Kuipers, E. J.  van Leerdam, M. E.  van Ballegooijen, M.  Stoker, J.  Dekker, E.  Steyerberg, E. W. | Endoscopy | 2013 | Time requirements and health effects of participation in colorectal cancer screening with colonoscopy or computed tomography colonography in a randomized controlled trial | Included |

An overview of each study’s author(s), publication journal, year of publication, title, and whether the study was included in the present analysis

# **Appendix F(b): SRs identified and assessed at study level (included and excluded)**

| **No.** | **Author** | **Journal** | **Year** | **Title** | **Note** |
| --- | --- | --- | --- | --- | --- |
| 1 | Brenner, H. Stock, C. Hoffmeister, M. | British Medical Journal | 2014 | Effect of screening sigmoidoscopy and screening colonoscopy on colorectal cancer incidence and mortality: systematic review and meta-analysis of randomised controlled trials and observational studies | Excluded: No harms investigated |
| 2 | Chandan, S.  Facciorusso, A.  Yarra, P.  Khan, S. R.  Ramai, D.  Mohan, B. P.  Kassab, L. L.  Bilal, M.  Shaukat, A. | American Journal of Gastroenterology | 2022 | Colonoscopy-Related Adverse Events in Patients With Abnormal Stool-Based Tests: A Systematic Review of Literature and Meta-analysis of Outcomes | Included |
| 3 | Day, L. W.  Kwon, A. Inadomi, J. M. Walter, L. C. Somsouk, M. | Gastrointestinal Endoscopy | 2011 | Adverse events in older patients undergoing colonoscopy: a systematic review and meta-analysis | Excluded: Mixed setting of endoscopy (i.e. screening, diagnostic, or therapeutic purposes) - data not stratified |
| 4 | Fitzpatrick-Lewis, D.  Ali, M. U.  Warren, R.  Kenny, M.  Sherifali, D.  Raina, P. | Clinical Colorectal Cancer | 2016 | Screening for Colorectal Cancer: A Systematic Review and Meta-Analysis | Included |
| 5 | Hewitson, P.  Glasziou, P.  Irwig, L.  Towler, B.  Watson, E. | Cochrane Database of Systematic Reviews | 2007 | Screening for colorectal cancer using the faecal occult blood test, Hemoccult | Included |
| 6 | Hofmann, B. | Journal of Evaluation in Clinical Practice | 2017 | Ethical issues with colorectal cancer screening-a systematic review | Excluded: Outside scope, focusing on ethical issues related to colorectal cancer screening, only including two studies reporting harms from screening |
| 7 | Holme, O.  Bretthauer, M.  Fretheim, A.  Odgaard-Jensen, J.  Hoff, G. | Cochrane Database of Systematic Reviews | 2013 | Flexible sigmoidoscopy versus faecal occult blood testing for colorectal cancer screening in asymptomatic individuals | Included |
| 8 | Huffstetler, A. N.  Fraiman, J.  Brownlee, S.  Stoto, M. A.  Lin, K. W. | Journal of the American Board of Family Medicine | 2023 | An Estimate of Severe Harms Due to Screening Colonoscopy: A Systematic Review | Included |
| 9 | Jaruvongvanich, V. Prasitlumkum, N. Assavapongpaiboon, B. Suchartlikitwong, S. Sanguankeo, A. Upala, S. | International Journal of Colorectal Disease | 2017 | Risk factors for delayed colonic post-polypectomy bleeding: a systematic review and meta-analysis | Excluded: Mixed setting of endoscopy (i.e. screening, diagnostic, or therapeutic purposes) - data not stratified |
| 10 | Jodal, H. C.  Helsingen, L. M.  Anderson, J. C.  Lytvyn, L.  Vandvik, P. O.  Emilsson, L. | British Medical Journal Open | 2019 | Colorectal cancer screening with faecal testing, sigmoidoscopy or colonoscopy: a systematic review and network meta-analysis | Included |
| 11 | Kayal, G.  Kerrison, R.  Hirst, Y.  von Wagner, C. | British Medical Journal Open | 2023 | Patients' experience of using colonoscopy as a diagnostic test after a positive FOBT/FIT: a systematic review of the quantitative literature | Included |
| 12 | Kindt, I. S.  Martiny, F. H. J.  Gram, E. G.  Bie, A. K. L.  Jauernik, C. P.  Rahbek, O. J.  Nielsen, S. B.  Siersma, V.  Bang, C. W.  Brodersen, J. B. | PLOS One | 2023 | The risk of bleeding and perforation from sigmoidoscopy or colonoscopy in colorectal cancer screening: A systematic review and meta-analyses | Included |
| 13 | Kothari, S. T. Huang, R. J. Shaukat, A. Agrawal, D. Buxbaum, J. L. Abbas Fehmi, S. M. Fishman, D. S. Gurudu, S. R. Khashab, M. A. Jamil, L. H. Jue, T. L. Law, J. K. Lee, J. K. Naveed, M. Qumseya, B. J. Sawhney, M. S. Thosani, N. Yang, J. DeWitt, J. M. Wani, S. | Gastrointestinal Endoscopy | 2019 | ASGE review of adverse events in colonoscopy | Excluded: Mixed setting of endoscopy (i.e. screening, diagnostic, or therapeutic purposes) - data not stratified |
| 14 | Lin, J. S.  Piper, M. A.  Perdue, L. A.  Rutter, C. M.  Webber, E. M.  O'Connor, E.  Smith, N.  Whitlock, E. P. | JAMA | 2016 | Screening for Colorectal Cancer: Updated Evidence Report and Systematic Review for the US Preventive Services Task Force | Included |
| 15 | Lin, J. S.  Perdue, L. A.  Henrikson, N. B.  Bean, S. I.  Blasi, P. R. | JAMA | 2021 | Screening for Colorectal Cancer: Updated Evidence Report and Systematic Review for the US Preventive Services Task Force | Included |
| 16 | Martiny, F. H. J.  Bie, A. K. L.  Jauernik, C. P.  Rahbek, O. J.  Nielsen, S. B.  Gram, E. G.  Kindt, I.  Siersma, V.  Bang, C. W.  Brodersen, J. B. | PLOS One | 2024 | Deaths and cardiopulmonary events following colorectal cancer screening-A systematic review with meta-analyses | Included |
| 17 | Niv, Y.  Hazazi, R.  Levi, Z.  Fraser, G. | Digestive Diseases and Sciences | 2008 | Screening colonoscopy for colorectal cancer in asymptomatic people: a meta-analysis | Included |
| 18 | Papastergiou, V. Mathou, N. Manes, K. Maniatis, P. Paraskeva, K. Evgenidi, A. Giannakopoulos, A. Karagiannis, J. A. | Acta Gastro-Enterologica Belgica | 2018 | When perforation is not the culprit : case report and systematic review of mechanical small-bowel obstruction complicating colonoscopy | Excluded: Mixed setting of endoscopy (i.e. screening, diagnostic, or therapeutic purposes) - data not stratified |
| 19 | Reumkens, A.  Rondagh, E. J.  Bakker, C. M.  Winkens, B.  Masclee, A. A.  Sanduleanu, S. | American Journal of Gastroenterology | 2016 | Post-Colonoscopy Complications: A Systematic Review, Time Trends, and Meta-Analysis of Population-Based Studies | Included |
| 20 | Steffenssen, M. W. Al-Najami, I. Baatrup, G. | Acta Oncologica | 2019 | Patient-reported minor adverse events after colonoscopy: a systematic review | Excluded: Mixed setting of endoscopy (i.e. screening, diagnostic, or therapeutic purposes) - data not stratified |
| 21 | Tinmouth, J.  Vella, E. T.  Baxter, N. N.  Dube, C.  Gould, M.  Hey, A.  Ismaila, N.  McCurdy, B. R.  Paszat, L. | Canadian Journal of Gastroenterology and Hepatology | 2016 | Colorectal Cancer Screening in Average Risk Populations: Evidence Summary | Included |
| 22 | Towler, B.  Irwig, L.  Glasziou, P.  Kewenter, J.  Weller, D.  Silagy, C. | British Medical Journal | 1998 | A systematic review of the effects of screening for colorectal cancer using the faecal occult blood test, hemoccult | Included |
| 23 | Vermeer, N. C.  Snijders, H. S.  Holman, F. A.  Liefers, G. J.  Bastiaannet, E.  van de Velde, C. J.  Peeters, K. C. | Cancer Treatment Reviews | 2017 | Colorectal cancer screening: Systematic review of screen-related morbidity and mortality | Included |
| 24 | Whitlock, E. P. Lin, J. S. Liles, E. Beil, T. L. Fu, R. | Annals of Internal Medicine | 2008 | Screening for colorectal cancer: a targeted, updated systematic review for the U.S. Preventive Services Task Force | Excluded: Updated study is included |

An overview of each study’s author(s), publication journal, year of publication, title, and whether the study was included in the present analysis

| **Appendix G Table 5a: Study- and outcome coverage of the RCTs:** | **Bleeding** | **Perforation** | **Pain** | **Complications/adverse events in total** | **Other harms** | **Cardiovascular - and pulmonary complications** | **Death** | **Discomfort** | **Colorectal symptoms** | **Complications related to bowel preparation** | **Inflammatory complications** | **Post-polypectomy syndrome** | **Nausea/vomiting** | **Infections** | **Sleep disturbances** | **Dizziness** | **Sedation-related complications** | **SC** |
| --- | --- | --- | --- | --- | --- | --- | --- | --- | --- | --- | --- | --- | --- | --- | --- | --- | --- | --- |
| **Senore 2011** | X |  | X | X | X | X |  |  | X | X |  |  |  |  |  | X |  | 8/17 |
| **Segnan 2002** | X | X | X | X | X | X |  | X |  |  | X |  |  |  |  |  |  | 8/17 |
| **Bretthauer 2016** | X | X | X | X | X | X | X |  |  |  |  | X |  |  |  |  |  | 8/17 |
| **Hol 2010b** | X |  | X |  | X |  |  | X | X | X |  |  | X |  |  |  |  | 7/17 |
| **Stoop 2012** | X |  | X | X | X | X | X |  |  |  | X |  |  | X |  |  |  | 7/17 |
| **Atkin 2002** | X | X | X |  |  | X | X |  |  |  | X |  |  |  |  |  |  | 6/17 |
| **Gondal 2003** | X | X |  |  | X |  |  |  |  | X |  | X |  |  |  |  |  | 5/17 |
| **Robinson 1999** | X | X |  | X | X |  | X |  |  |  |  |  |  |  |  |  |  | 5/17 |
| **Wijkerslooth 2012** |  |  | X |  | X |  |  |  | X | X |  |  |  |  | X |  |  | 5/17 |
| **Forbes 2006** | X | X | X | X |  |  |  |  |  |  |  |  |  |  |  |  |  | 4/17 |
| **Quintero 2012** | X | X |  | X |  | X |  |  |  |  |  |  |  |  |  |  |  | 4/17 |
| **Van Dam 2013** |  |  |  |  | X | X |  |  | X |  |  |  | X |  |  |  |  | 4/17 |
| **Holme 2014** | X | X |  | X |  |  | X |  |  |  |  |  |  |  |  |  |  | 4/17 |
| **Segnan 2005** | X |  | X |  |  | X |  |  |  |  |  |  |  |  |  |  |  | 3/17 |
| **Fritzell 2020** |  |  | X | X |  |  |  | X |  |  |  |  |  |  |  |  |  | 3/17 |
| **Randel 2021** | X | X |  |  |  |  | X |  |  |  |  |  |  |  |  |  |  | 3/17 |
| **Kewenter 1996** | X | X |  | X |  |  |  |  |  |  |  |  |  |  |  |  |  | 3/17 |
| **Hol 2010a** | X |  |  |  | X |  |  |  |  |  |  |  |  |  |  |  |  | 2/17 |
| **Larsen 2009** |  |  | X |  |  |  |  | X |  |  |  |  |  |  |  |  |  | 2/17 |
| **Mandel 1993** | X | X |  |  |  |  |  |  |  |  |  |  |  |  |  |  |  | 2/17 |
| **Kobiela 2019** |  |  |  |  | X |  | X |  |  |  |  |  |  |  |  |  |  | 2/17 |
| **Hoff 2009** |  |  |  | X |  |  |  |  |  |  |  |  |  |  |  |  |  | 1/17 |
| **Rasmussen 1999** |  |  | X | X |  |  |  |  |  |  |  |  |  |  |  |  |  | 1/17 |
| **Schoen 2012** |  | X |  |  |  |  |  |  |  |  |  |  |  |  |  |  |  | 1/17 |
| **OC** | 16/24 | 12/24 | 12/24 | 12/24 | 11/24 | 8/24 | 7/24 | 4/24 | 4/24 | 4/24 | 3/24 | 2/24 | 2/24 | 1/24 | 1/24 | 1/24 | 0/24 |  |

*Table 5a. Study coverage (SC) and outcome coverage (OC) of all 24 included RCTs*

# **Appendix H: Other harms**

| **All outcomes categorised as “Other harms” in the reference review** |
| --- |
| Other (not defined) (IV*) |
| Incontinence (II) |
| Other major complications |
| Acute urinary retention |
| Other moderate and severe adverse events (ASGE**) |
| Moderate and severe adverse events (ASGE) |
| Serious adverse events (NIH***) |
| Incidents |
| Miscellaneous |
| "Most burdensome part of interventions" / Most burdensome aspect of the overall screening procedure |
| Post Perforation Morbidity defined as an in-patient complication or new diagnosis |
| Paralytic ileus |
| “During the colonoscopy I asked for it to be stopped or paused” |
| Serious complications |
| Fever (II) |
| Minor events (including vasovagal reactions) not requiring hospitalization |
| Burden |
| Complications: bleeding or pain |
| Weight loss, fatigue and diarrhoea for several months |
| "Other unplanned events, minor: Procedure aborted, unplanned post procedure medical consultation, unplanned admission or prolongation of hospital stay of <3 nights, use of reversal agent, hypoxia (sat <85%) or hypotension (BP <90/50)" |
| "Other unplanned events, intermediate: Unplanned admission or prolongation of hospital stay of >10 nights or ITU admission >1 night, Intervention - endoscopic or radiological, Interventional treatment for skin or other tissue injury, Unplanned ventilatory support during conscious sedation" |
| "Other unplanned events - major: Surgery for adverse event/sequelae, Permanent disability, Unplanned admission or prolongation of hospital stay of >10 nights or ITU admission >1 night" |
| "Other unplanned events, fatal: death" |
| Hospitalizations (III) |
| Lesion during colonoscopy |
| Any other complication requiring hospital admission (II) |
| Self-rated health |
| Other comments about any aspects of the test |
| Snare entrapment (II) |
| Emergency department and urgent care visits. |
| Re-admissions: Fall, anaemia (gastroscopy negative) |
| Re-admissions: Headache, nausea, vomiting (CT negative) |
| Re-admissions: Joint pain and anaemia |
| Re-admissions: Migraine |
| Re-operations: fever abdominal pain, laparoscopy |
| Re-operations: colonic obstruction, colostomy |
| Re-operations: inguinal hernia with perforation in diverticulum |
| Epileptic seizure requiring medical treatment |
| Minor self-limited complications |
| Rectal prolapse leading to hospital admission within 30 days |
| Abdominal hernia leading to hospital admission within 30 days |
| Orthopaedic surgery (hospital admission within 30 days |
| "Other" leading to hospital admission within 30 days |
| Anal irritation |
| "Other AEs (including unspecified complications of surgical and medical care as well as anaesthesiologic complications)" |
| Collapse |
| Ingestion of disinfectant |
| Minor adverse events |
| Physical functioning |
| Returning to routines after examination |
| Recovery time after colonoscopy |
| Feeling back to normal after examination |
| Tiredness |
| Headache |
| Hindered in normal activities |
| Seizure |
| Trouble sleeping |
| Peritonitis like reaction requiring hospitalization |
| Hernia |
| Major morbidity after colonoscopy |
| * Roman numerals specify the amount of times the result have been reported ** ASGE: American society for gastrointestinal endoscopy *** NIH: National Institutes of Health |

# **Appendix I Table 5b: Study- and outcome coverage of the SRs**

|  | **Perforation** | **Bleeding** | **Death** | **Cardiovascular - and pulmonary complications** | **Other harms** | **Complications/adverse events in total** | **Post-polypectomy syndrome** | **Pain** | **Inflammatory complications** | **Discomfort** | **Colorectal symptoms** | **Sedation-related complications** | **Infections** | **Complications related to bowel preparation** | **Sleep disturbances** | **Nausea/vomiting** | **Dizziness** | **SC** |
| --- | --- | --- | --- | --- | --- | --- | --- | --- | --- | --- | --- | --- | --- | --- | --- | --- | --- | --- |
| **Lin 2016** | X | X | X | X | X |  |  | X | X |  | X |  | X |  |  |  |  | 9/17 |
| **Jodal 2019** | X | X | X | X | X | X | X | X |  |  |  |  |  |  |  |  |  | 8/17 |
| **Vermeer 2017** | X | X | X | X |  | X | X | X |  | X |  |  |  |  |  |  |  | 8/17 |
| **Chandan 2022** | X | X | X | X | X | X | X |  |  |  |  | X |  |  |  |  |  | 8/17 |
| **Kayal 2023** | X | X |  | X | X |  | X | X |  | X |  |  |  | X |  |  |  | 8/17 |
| **Holme 2013** | X | X | X | X | X | X |  |  | X |  |  |  |  |  |  |  |  | 7/17 |
| **Hewitson 2007** | X | X | X |  | X | X |  |  | X |  |  |  |  |  |  |  |  | 6/17 |
| **Reumkens 2016** | X | X | X |  |  |  |  |  |  |  |  |  |  |  |  |  |  | 3/17 |
| **Fitzpatrick-Lewis 2016** | X | X | X |  |  |  |  |  |  |  |  |  |  |  |  |  |  | 3/17 |
| **Niv 2008** | X | X | X |  |  |  |  |  |  |  |  |  |  |  |  |  |  | 3/17 |
| **Lin 2021** | X | X |  |  | X |  |  |  |  |  |  |  |  |  |  |  |  | 3/17 |
| **Huffstetler 2023** | X | X |  | X |  |  |  |  |  |  |  |  |  |  |  |  |  | 3/17 |
| **Kindt 2023** | X | X |  |  |  |  |  |  |  |  |  |  |  |  |  |  |  | 3/17 |
| **Towler 1998** | X | X |  |  |  |  |  |  |  |  |  |  |  |  |  |  |  | 2/17 |
| **Martiny 2024** |  |  | X | X |  |  |  |  |  |  |  |  |  |  |  |  |  | 2/17 |
| **Tinmouth 2016** |  |  |  |  |  | X |  |  |  |  |  |  |  |  |  |  |  | 1/17 |
| **OC** | 14/16 | 14/16 | 10/16 | 8/16 | 7/16 | 6/16 | 4/16 | 4/16 | 3/16 | 2/16 | 1/16 | 1/16 | 1/16 | 0 | 0 | 0 | 0 |  |

*Table 5b. Study coverage (SC) and outcome coverage (OC) of all 16 included SRs*

1. With the exception of “other (not defined)” and “hospitalisations” which we deemed too unspecific and placed in the category “Other harms” (appendix H). [↑](#footnote-ref-1)
