# Supplementary material for: Physical harms in colorectal cancer screening: An overview of the reporting in systematic reviews and randomised controlled trials
Source: PLoS One. 2025 Sep 11;20(9):e0331104. doi: 10.1371/journal.pone.0331104 (PMC12425235; doi:10.1371/journal.pone.0331104)
Supplement: S2 File — S2 includes Appendices J and K, which contain all extracted outcomes from RCTs and SRs, respectively. (DOCX) [file pone.0331104.s002.docx]

# **S2: Appendices J and K Tables presenting all extracted data from randomised controlled trials and systematic reviews/studies, respectively.** All studies fulfilled the inclusion criteria for the review.

# **Appendix J Table 6. Extracted data from included RCTs.**

| **Study-ID** | **Definition made in study** | **Type of harm** | **Severity assessment** |
| --- | --- | --- | --- |
| Bretthauer 2016 | Major bleeding, owing to polypectomy | Bleeding | Severe |
| Bretthauer 2016 | Vasovagal reaction without need of extra measures | Cardiovascular- and pulmonary complications | Not Severe |
| Bretthauer 2016 | Death | Death | Death |
| Bretthauer 2016 | Other major complications | Other harms | Severe |
| Bretthauer 2016 | Abdominal pain | Pain | Not Severe |
| Bretthauer 2016 | Perforation leading to laparotomy | Perforation | Very Severe |
| Bretthauer 2016 | Polypectomy serosal burns (no intervention necessary) | Post-polypectomy syndrome | Severe |
| Robinson 1999 | Major bleeding, treated endoscopically | Bleeding | Severe |
| Robinson 1999 | Complications/adverse events in total, requiring surgical intervention. | Complications/Adverse events in total | Very Severe |
| Robinson 1999 | Complications/adverse events in total, not requiring surgical intervention. | Complications/Adverse events in total | Not Severe |
| Robinson 1999 | Death | Death | Death |
| Robinson 1999 | Snare entrapment | Other harms | Unknown |
| Robinson 1999 | Perforation | Perforation | Severe |
| Larsen 2002 | Discomfort from endoscopy examination (headache, nausea, fatigue, general pain) | Discomfort | Not Severe |
| Larsen 2002 | Discomfort from endoscopy examination (flatulence, diarrhoea, abdominal aches, pain) | Discomfort | Not Severe |
| Larsen 2002 | Pain during examination: slightly | Pain | Not Severe |
| Larsen 2002 | Pain during examination: moderately | Pain | Not Severe |
| Larsen 2002 | Pain during examination: very | Pain | Severe |
| Mandel 1993 | Serious bleeding requiring surgery | Bleeding | Very Severe |
| Mandel 1993 | Serious bleeding not requiring surgery | Bleeding | Severe |
| Mandel 1993 | Perforations, all requiring surgery | Perforation | Very Severe |
| Hoff 2009 | Severe complications | Complications/Adverse events in total | Severe |
| Van Dam 2013 | Vasovagal syncope | Cardiovascular- and pulmonary complications | Not Severe |
| Van Dam 2013 | Abdominal complaints | Colorectal symptoms | Unknown |
| Van Dam 2013 | Nausea | Nausea/vomiting | Not Severe |
| Van Dam 2013 | Feeling back to normal after examination | Other harms | Not Severe |
| Van Dam 2013 | Returning to routines after examination | Other harms | Unknown |
| Van Dam 2013 | Recovery time after colonoscopy | Other harms | Unknown |
| Van Dam 2013 | Tiredness | Other harms | Not Severe |
| Van Dam 2013 | Headache | Other harms | Not Severe |
| Stoop 2012 | Post-polypectomy bleeding | Bleeding | Unknown |
| Stoop 2012 | Acute coronary syndrome | Cardiovascular- and pulmonary complications | Very Severe |
| Stoop 2012 | Cerebrovascular accident | Cardiovascular-and pulmonary complications | Very Severe |
| Stoop 2012 | Myocardial infarction | Cardiovascular-and pulmonary complications | Very Severe |
| Stoop 2012 | Atrial fibrillation | Cardiovascular-and pulmonary complications | Severe |
| Stoop 2012 | Death | Death | Death |
| Stoop 2012 | Pneumonia | Infections | Unknown |
| Stoop 2012 | Spinal epidural abscess | Infections | Unknown |
| Stoop 2012 | Urinary tract infection | Infections | Unknown |
| Stoop 2012 | Acute rheumatic fever | Inflammatory complications | Severe |
| Stoop 2012 | Ingestion of disinfectant | Other harms | Unknown |
| Stoop 2012 | Collapse | Other harms | Unknown |
| Stoop 2012 | Pain causing incomplete procedure | Pain | Severe |
| Wijkerslooth 2012 | Burdensome (including abdominal symptoms afterwards); somewhat, rather, extremely | Colorectal symptoms | Unknown |
| Wijkerslooth 2012 | Perceived pain during bowel preparation | Complications related to bowel preparation | Not Severe |
| Wijkerslooth 2013 | Trouble sleeping | Other harms | Unknown |
| Wijkerslooth 2012 | Most burdensome aspect of the overall screening procedure | Other harms | Not Severe |
| Wijkerslooth 2013 | Hindered in normal activities | Other harms | Unknown |
| Wijkerslooth 2012 | Abdominal complaints (more than normal) after examination, perceived as being painful | Pain | Not Severe |
| Wijkerslooth 2012 | Pain (somewhat) | Pain | Not Severe |
| Wijkerslooth 2012 | Pain (rather) | Pain | Not Severe |
| Wijkerslooth 2012 | Pain (extremely) | Pain | Severe |
| Wijkerslooth 2012 | Painful to some degree | Pain | Not Severe |
| Hol 2010a (2009) | Minimal rectal bleeding | Bleeding | Not Severe |
| Hol 2010a (2009) | Colovaginal fistula due to a previous diverticulitis. It was assumed that the air insufflation led to symptoms, since no diverticulitis was seen during examination. | Infections | Very Severe |
| Hol 2010b | Collecting faeces (quite) | Discomfort | Not Severe |
| Hol 2010b | Collecting faeces (very) | Discomfort | Not Severe |
| Hol 2010b | Performing the test (quite) | Discomfort | Not Severe |
| Hol 2010b | Performing the test (very) | Discomfort | Not Severe |
| Hol 2010b | Returning the test (quite) | Discomfort | Not Severe |
| Hol 2010b | Returning the test (very) | Discomfort | Not Severe |
| Hol 2010b | Rectal blood loss | Bleeding | Unknown |
| Hol 2010b | Faecal incontinence | Colorectal symptoms | Unknown |
| Hol 2010b | Constipation | Colorectal symptoms | Not Severe |
| Hol 2010b | Flatulence or feeling bloated | Colorectal symptoms | Not Severe |
| Hol 2010b | Diarrhoea | Colorectal symptoms | Not Severe |
| Hol 2010b | Pain (during preparation) | Complications related to bowel preparation | Not Severe |
| Hol 2010b | Discomfort (during preparation) | Complications related to bowel preparation | Not Severe |
| Hol 2010b | Discomfort (quite) | Discomfort | Not Severe |
| Hol 2010b | Discomfort (very) | Discomfort | Not Severe |
| Hol 2010b | Discomfort directly afterwards | Discomfort | Not Severe |
| Hol 2010b | Discomfort related to sigmoidoscopy | Discomfort | Not Severe |
| Hol 2010b | Nausea and vomiting | Nausea/vomiting | Not Severe |
| Hol 2010b | Burden | Other harms | Unknown |
| Hol 2010b | Anal pain | Pain | Not Severe |
| Hol 2010b | Pain during sigmoidoscopy | Pain | Not Severe |
| Hol 2010b | Pain directly afterwards | Pain | Not Severe |
| Hol 2010b | Abdominal pain | Pain | Not Severe |
| Hol 2010b | Very painful | Pain | Severe |
| Quintero 2012 | Bleeding | Bleeding | Unknown |
| Quintero 2012 | Desaturation | Cardiovascular-and pulmonary complications | Not Severe |
| Quintero 2012 | Hypotension or bradycardia | Cardiovascular-and pulmonary complications | Not Severe |
| Quintero 2012 | Perforation, bleeding, desaturation, hypotension or bradycardia | Complications/Adverse events in total | Unknown |
| Quintero 2012 | Perforation | Perforation | Severe |
| Atkin 2002 | Admitted to hospital for bleeding: treated conservatively | Bleeding | Severe |
| Atkin 2002 | Admitted to hospital for bleeding: requiring surgical suture | Bleeding | Very Severe |
| Atkin 2002 | Admitted to hospital for bleeding: requiring injection of adrenaline at the polypectomy site | Bleeding | Severe |
| Atkin 2002 | Bleeding starting within 1 month and lasting longer than 4 days | Bleeding | Unknown |
| Atkin 2002 | Non-fatal myocardial infarctions | Cardiovascular-and pulmonary complications | Very Severe |
| Atkin 2002 | Pulmonary embolism | Cardiovascular-and pulmonary complications | Very Severe |
| Atkin 2002 | Fainted or had a vasovagal episode | Cardiovascular-and pulmonary complications | Not Severe |
| Atkin 2002 | Death | Death | Death |
| Atkin 2002 | Glutaraldehyde-induced colitis | Inflammatory complications | Severe |
| Atkin 2002 | Mild pain | Pain | Not Severe |
| Atkin 2002 | Severe pain | Pain | Severe |
| Atkin 2002 | Perforation | Perforation | Severe |
| Senore 2011 | Bleeding | Bleeding | Unknown |
| Senore 2011 | Cardiopulmonary events resulting in hospital admissions within 30 days | Cardiovascular-and pulmonary complications | Severe |
| Senore 2011 | Other GI tract symptoms leading to hospital admission within 30 days | Colorectal symptoms | Severe |
| Senore 2011 | Alterations of bowel habits | Colorectal symptoms | Not Severe |
| Senore 2011 | Bowel distension | Colorectal symptoms | Not Severe |
| Senore 2011 | Nausea (due to bowel preparation) | Complications related to bowel preparation | Not Severe |
| Senore 2011 | Faint/dizzy (due to bowel preparation) | Complications related to bowel preparation | Not Severe |
| Senore 2011 | Other symptoms (in relation to bowel preparation) | Complications related to bowel preparation | Unknown |
| Senore 2011 | Anal irritation (due to bowel preparation) | Complications related to bowel preparation | Not Severe |
| Senore 2011 | Bowel distension (due to bowel preparation) | Complications related to bowel preparation | Not Severe |
| Senore 2011 | Incontinence (due to bowel preparation) | Complications related to bowel preparation | Unknown |
| Senore 2011 | Abdominal pain (due to bowel preparation) | Complications related to bowel preparation | Not Severe |
| Senore 2011 | Faint/dizzy | Dizziness | Not Severe |
| Senore 2011 | Vomiting | Nausea/vomiting | Not Severe |
| Senore 2011 | Rectal prolapse leading to hospital admission within 30 days | Other harms | Severe |
| Senore 2011 | Abdominal hernia leading to hospital admission within 30 days | Other harms | Severe |
| Senore 2011 | Orthopaedic surgery (hospital admission within 30 days | Other harms | Severe |
| Senore 2011 | "Other" leading to hospital admission within 30 days | Other harms | Severe |
| Senore 2011 | Other symptoms | Other harms | Unknown |
| Senore 2011 | Anal irritation | Other harms | Not Severe |
| Senore 2011 | Incontinence | Other harms | Unknown |
| Senore 2011 | Pain | Pain | Not Severe |
| Senore 2011 | Hypotension | Cardiovascular-and pulmonary complications | Not Severe |
| Senore 2011 | Late post-procedural reactions (adverse physical reactions following discharge): Bowel distension, pain, anal irritation, dizziness, bleeding, alteration of bowel habits, incontinence, other symptoms | Complications/Adverse events in total | Unknown |
| Senore 2011 | Pain assessment item: The most severe pain I ever experienced | Pain | Severe |
| Senore 2011 | Pain assessment item: I hope it will not be necessary to repeat the test | Pain | Not Severe |
| Segnan 2002 | Bleeding following polypectomy requiring hospitalization | Bleeding | Severe |
| Segnan 2002 | Self-limiting bleeding following polypectomy | Bleeding | Not Severe |
| Segnan 2002 | Allergic reaction to latex gloves | Inflammatory complications | Unknown |
| Segnan 2002 | Glutaraldehyde colitis | Inflammatory complications | Severe |
| Segnan 2002 | Epileptic seizure requiring medical treatment | Other harms | Severe |
| Segnan 2002 | Peritonitis like reaction requiring hospitalization | Other harms | Severe |
| Segnan 2002 | Minor self-limited complications | Other harms | Not Severe |
| Segnan 2002 | Pain | Pain | Not Severe |
| Segnan 2002 | Procedural pain (The most severe pain I ever experienced) | Pain | Severe |
| Segnan 2002 | Perforation requiring hospitalization | Perforation | Severe |
| Segnan 2002 | Mild vagal reaction (nausea, feeling faint, dizzy) | Cardiovascular-and pulmonary complications | Not Severe |
| Segnan 2002 | Complications requiring hospitalization | Complications/Adverse events in total | Severe |
| Segnan 2002 | Mild discomfort | Discomfort | Not Severe |
| Segnan 2002 | Abdominal pain | Pain | Not Severe |
| Segnan 2005 | Severe haemorrhage requiring hospitalization | Bleeding | Severe |
| Segnan 2005 | Self-limited bleeding following polypectomy | Bleeding | Not Severe |
| Segnan 2005 | Mild vagal reaction (nausea, feeling faint, dizzy) | Cardiovascular-and pulmonary complications | Not Severe |
| Segnan 2005 | The most severe pain I ever experienced | Pain | Severe |
| Segnan 2005 | Abdominal pain | Pain | Not Severe |
| Segnan 2005 | Mild pain | Pain | Not Severe |
| Segnan 2005 | Severe vagal reaction and cardiac arrest (resuscitated and subsequently discharged from the hospital within 24 hours) | Cardiovascular-and pulmonary complications | Very Severe |
| Gondal 2003 | Minor events (including vasovagal reactions) | Complications related to bowel preparation | Not Severe |
| Gondal 2003 | Serious adverse events | Other harms | Severe |
| Gondal 2003 | Postpolypectomy bleeding: not requiring intervention or transfusion | Bleeding | Not Severe |
| Gondal 2003 | Fever | Other harms | Not Severe |
| Gondal 2003 | Minor events (including vasovagal reactions) not requiring hospitalization | Other harms | Not Severe |
| Gondal 2003 | Perforation resulting in hemicolectomy | Perforation | Very Severe |
| Gondal 2003 | Perforation resulting in local suture at laparoscopy or laparotomy | Perforation | Very Severe |
| Gondal 2003 | Burnt serosa syndrome | Post-polypectomy syndrome | Severe |
| Forbes 2006 | Bleeding | Bleeding | Unknown |
| Forbes 2006 | Other serious complications | Complications/Adverse events in total | Severe |
| Forbes 2006 | Pain | Pain | Not Severe |
| Forbes 2006 | Perforation | Perforation | Severe |
| Rasmussen 1999 | Serious complications | Complications/Adverse events in total | Severe |
| Schoen 2012 | Perforation | Perforation | Severe |
| Kewenter 1996 | Bleeding | Bleeding | Unknown |
| Kewenter 1996 | Heavy intestinal bleeding leading to readmission and surgical intervention | Bleeding | Very Severe |
| Kewenter 1996 | Perforation due to polypectomy leading to surgical intervention | Perforation | Very Severe |
| Fritzell 2020 | “To what extent did you feel discomfort during the examination?” (Not at all/Fairly little/Neither nor/A lot/Very much) | Discomfort | Not Severe |
| Fritzell 2020 | “To what extent did you feel pain during the examination?” (Not at all/Fairly little/Neither nor/A lot/Very much) | Pain | Severe |
| Fritzell 2020 | Complications | Complications/Adverse events in total | Unknown |
| Holme 2014 | Death | Death | Death |
| Holme 2014 | Polypectomy bleeding following snare polypectomy | Bleeding | Severe |
| Holme 2014 | Perforation | Perforation | Severe |
| Holme 2014 | Complications | Complications/Adverse events in total | Unknown |
| Kobiela 2019 | Death | Death | Death |
| Kobiela 2019 | Hospitalization | Other harms | Severe |
| Randel 2021 | Death | Death | Death |
| Randel 2021 | Significant bleedings | Bleeding | Severe |
| Randel 2021 | Perforations (“6 were conservatively treated with antibiotics.") | Perforation | Severe |
| Randel 2021 | Perforations (“One of the perforations was surgically treated”) | Perforation | Very Severe |

***Table 6*** *presents all extracted outcomes as defined in the original publications, the type of harm as categorized by authors AKB and FM, and the corresponding severity assessments.*

# **Appendix K Table 6. Extracted data from included SRs.**

| **Study-ID** | **Definition made in study** | **Type of harm** | **Severity assessment** |
| --- | --- | --- | --- |
| Chandan 2022 | Death | Death | Death |
| Chandan 2022 | Bleeding (minor, intermediate) | Bleeding | Not Severe |
| Chandan 2022 | Bleeding (Serious/Severe) | Bleeding | Severe |
| Chandan 2022 | Bleeding (postpolypectomy bleeding (involving transfusion or hospitalization of at least 24 hours), Serious/severe bleeding requiring surgery, Bleeding requiring hospitalization | Bleeding | Very Severe |
| Chandan 2022 | Bleeding | Bleeding | Unknown |
| Chandan 2022 | Perforation | Perforation | Severe |
| Chandan 2022 | Perforation (all requiring surgery) | Perforation | Very Severe |
| Chandan 2022 | Cardiopulmonary events | Cardiovascular – and pulmonary complications | Unknown |
| Chandan 2022 | Postpolypectomy syndrome | Postpolypectomy syndrome | Unknown |
| Chandan 2022 | Sedation related (adverse events) | Sedation-related complications | Unknown |
| Chandan 2022 | Severe adverse events requiring surgical intervention and/or hospitalization. Pooled incidence of perforation, bleeding and postpolypectomy syndrome. | Complications/Adverse events in total | Severe |
| Fitzpatrick-Lewis 2016 | Major bleeding requiring hospitalization | Bleeding | Severe |
| Fitzpatrick-Lewis 2016 | Minor bleeding (not requiring hospitalization | Bleeding | Not Severe |
| Fitzpatrick-Lewis 2016 | All-cause mortality | Death | Death |
| Fitzpatrick-Lewis 2016 | Death | Death | Death |
| Fitzpatrick-Lewis 2016 | Perforation | Perforation | Severe |
| Hewitzon 2011 | Bleeding detected 12 days after polypectomy | Bleeding | Unknown |
| Hewitzon 2011 | Serious bleeding | Bleeding | Severe |
| Hewitzon 2011 | Serious bleeding requiring surgery | Bleeding | Very Severe |
| Hewitzon 2011 | Major bleed | Bleeding | Severe |
| Hewitzon 2011 | Snare entrapment | Other harms | Unknown |
| Hewitzon 2011 | Perforation | Perforation | Severe |
| Hewitzon 2011 | Perforation of the colon requiring surgery | Perforation | Very Severe |
| Holme 2013 | Bleeding | Bleeding | Unknown |
| Holme 2013 | Major complications | Complications/Adverse events in total | Severe |
| Holme 2013 | Death within 30 days of procedure | Death | Death |
| Holme 2013 | Miscellaneous | Other harms | Unknown |
| Holme 2013 | Perforation | Perforation | Severe |
| Huffstetler 2023 | Severe bleeding requiring repeat endoscopic evaluation | Bleeding | Very Severe |
| Huffstetler 2023 | Severe postpolypectomy bleeding | Bleeding | Severe |
| Huffstetler 2023 | Severe bleeding requiring admission to the hospital | Bleeding | Severe |
| Huffstetler 2023 | Severe bleeding requiring transfusion of at least 1 unit of packed red blood cells, | Bleeding | Severe |
| Huffstetler 2023 | Cardiovascular adverse events | Cardiovascular - and pulmonary complications | Unknown |
| Huffstetler 2023 | Perforation (defined as free air or perforation visualized on radiograph requiring hospitalization) | Perforation | Severe |
| Huffstetler 2023 | Perforation (defined as free air or perforation visualized on radiograph requiring surgery) | Perforation | Very Severe |
| Jodal 2019 | Bleeding requiring hospitalisation | Bleeding | Severe |
| Jodal 2019 | Myocardial infarction | Cardiovascular-and pulmonary complications | Very Severe |
| Jodal 2019 | Pulmonary embolus | Cardiovascular-and pulmonary complications | Very Severe |
| Jodal 2019 | Hypotension or bradycardia | Cardiovascular-and pulmonary complications | Not Severe |
| Jodal 2019 | Desaturation | Cardiovascular-and pulmonary complications | Not Severe |
| Jodal 2019 | Major complications within 30 days of screening | Complications/Adverse events in total | Severe |
| Jodal 2019 | Miscellaneous within 30 days of screening or diagnostic workup: snare entrapment, vasovagal reactions, glutaraldehyde colitis and other events not requiring hospitalisation | Complications/Adverse events in total | Not Severe |
| Jodal 2019 | Death <30 days of procedure | Death | Death |
| Jodal 2019 | Death <30 days of surgery | Death | Death |
| Jodal 2019 | Fever | Other harms | Not Severe |
| Jodal 2019 | Procedure-related pain | Pain | Not Severe |
| Jodal 2019 | Procedure-related pain: moderate-severe pain | Pain | Not Severe |
| Jodal 2019 | Perforations after screening, either from the screening procedure itself or subsequent workup | Perforation | Severe |
| Jodal 2019 | Burnt serosa syndrome | Post-polypectomy syndrome | Severe |
| Kayal 2023 | Rectal bleeding | Bleeding | Unknown |
| Kayal 2023 | Post-plypectomy bleeding | Bleeding | Unknown |
| Kayal 2023 | Cardiac arrhytmias | Cardiovascular- and pulmonary complications | Unknown |
| Kayal 2023 | Respiratory arrest | Cardiovascular- and pulmonary complications | Very Severe |
| Kayal 2023 | Discomfort during preparation | Complications related to bowel preparation, | Not Severe |
| Kayal 2023 | The burden of drinking the bowel preparation | Complications related to bowel preparation, | Not Severe |
| Kayal 2023 | The burden of abdominal reports | Complications related to bowel preparation, | Not Severe |
| Kayal 2023 | Abdominal problems (post-procedure) | Discomfort | Not Severe |
| Kayal 2023 | More uncomfortable than expected (post-procedure) | Discomfort | Not Severe |
| Kayal 2023 | More uncomfortable than expected (during procedure) | Discomfort | Not Severe |
| Kayal 2023 | Pain and discomfort (during procedure) (only registered as pain) | Pain | Not Severe |
| Kayal 2023 | Pain and discomfort (post-procedure) (only registered as pain) | Pain | Not Severe |
| Kayal 2023 | High level of pain (post-procedure) | Pain | Severe |
| Kayal 2023 | Pain (postprocedure) | Pain | Not Severe |
| Kayal 2023 | Perforation | Perforation | Severe |
| Kayal 2023 | Colonic perforation | Perforation | Severe |
| Kayal 2023 | Post-polypectomy syndrome | Post-polypectomy syndrome | Unknown |
| Kayal 2023 | Time before resuming to normal activity | Other | Unknown |
| Kindt 2023 | …not requiring transfusion - We also identified all adverse events occurring within 30 days after baseline that were severe enough to require an emergency department visit or hospitalization. We classified adverse events as serious gastrointestinal (perforation, gastrointestinal bleeding requiring transfusion), other gastrointestinal bleeding not requiring transfusion, paralytic ileus, nausea, vomiting and dehydration, abdominal pain), or cardiovascular (myocardial infarction or angina, arrhythmias, congestive heart failure, cardiac or respiratory arrest, syncope, hypotension or shock) events (Subcategory: Bleeding). | Bleeding | Not Severe |
| Kindt 2023 | Self limiting (Subcategory: Bleeding) | Bleeding | Not Severe |
| Kindt 2023 | Requiring transfusion (Subcategory: Bleeding) | Bleeding | Severe |
| Kindt 2023 | Serious bleeding events | Bleeding | Severe |
| Kindt 2023 | Serious lower GI bleeding | Bleeding | Severe |
| Kindt 2023 | We defined significant bleedings as bleedings that lead to hospitalization (!1 day), blood transfusion, repeat endoscopy, radiologic intervention, or surgery | Bleeding | Unknown |
| Kindt 2023 | "admitted to the hospital for postpolypectomy bleeding following snare polypectomy." | Bleeding | Severe |
| Kindt 2023 | admitted to hospital for bleeding | Bleeding | Severe |
| Kindt 2023 | immediate hospitalization (Subcategory: Bleeding) | Bleeding | Severe |
| Kindt 2023 | Major bleeding | Bleeding | Severe |
| Kindt 2023 | Severe hemorrhage | Bleeding | Severe |
| Kindt 2023 | Bleedings | Bleeding | Unknown |
| Kindt 2023 | Bleeding resulting in need of transfusion | Bleeding | Severe |
| Kindt 2023 | Perforation resulting in death | Death | Death |
| Kindt 2023 | Perforation was defined as radiologic (computer tomography) findings consistent with intestinal perforation. | Perforation | Severe |
| Kindt 2023 | Acute complications (Subcategory: Perforations) | Perforation | Severe |
| Kindt 2023 | Causing hospitalizati–n - immediate complications (Subcategory: Perforations) | Perforation | Severe |
| Kindt 2023 | severe complications (Subcategory: Perforations) | Perforation | Severe |
| Kindt 2023 | Perforations | Perforation | Severe |
| Kindt 2023 | We also identified all adverse events occurring within 30 days after baseline that were severe enough to require an emergency department visit or hospitalization (Subcategory: Perforations) | Perforation | Severe |
| Kindt 2023 | Perforation resulting in treatment | Perforation | Severe |
| Kindt 2023 | Perforation resulting in morbidity | Perforation | Severe |
| Lin 2021 | Serious/Major bleeding | Bleeding | Severe |
| Lin 2021 | Perforation | Perforation | Severe |
| Lin 2021 | Other harms (cardiopulmonary events and infections) | Other harms | Unknown |
| Lin 2016 | Major bleeding | Bleeding | Severe |
| Lin 2016 | Cardiovascular events | Cardiovascular-and pulmonary complications | Unknown |
| Lin 2016 | Myocardial infarction | Cardiovascular-and pulmonary complications | Very Severe |
| Lin 2016 | Cerebrovascular accident | Cardiovascular-and pulmonary complications | Very Severe |
| Lin 2016 | Cerebrovascular disease | Cardiovascular-and pulmonary complications | Very Severe |
| Lin 2016 | Hypotension | Cardiovascular-and pulmonary complications | Not Severe |
| Lin 2016 | Syncope | Cardiovascular-and pulmonary complications | Not Severe |
| Lin 2016 | Pulmonary embolism | Cardiovascular-and pulmonary complications | Very Severe |
| Lin 2016 | Severe abdominal symptoms | Colorectal symptoms | Severe |
| Lin 2016 | Severe diarrhoea | Colorectal symptoms | Severe |
| Lin 2016 | Mortality | Death | Death |
| Lin 2016 | Colitis | Infections | Severe |
| Lin 2016 | Seizure | Other harms | Severe |
| Lin 2016 | Hernia | Other harms | Severe |
| Lin 2016 | Other serious events | Other harms | Unknown |
| Lin 2016 | Hospitalization | Other harms | Severe |
| Lin 2016 | Emergency department | Other harms | Unknown |
| Lin 2016 | Severe pain | Pain | Severe |
| Lin 2016 | Perforation | Perforation | Severe |
| Martiny 2023 | Cardiopulmonary events | Cardiovascular- and pulmonary complications | Unknown |
| Martiny 2023 | Acute coronary syndrome short-term and long-term | Cardiovascular- and pulmonary complications | Very Severe |
| Martiny 2023 | Arrhythmia short term and long-term | Cardiovascular- and pulmonary complications | Unknown |
| Martiny 2023 | Heart failure short-term and long-term | Cardiovascular- and pulmonary complications | Very Severe |
| Martiny 2023 | Pulmonary event short-term and long-term | Cardiovascular- and pulmonary complications | Unknown |
| Martiny 2023 | Stroke short-term and long-term | Cardiovascular- and pulmonary complications | Very Severe |
| Martiny 2023 | Tromboembolic event short-term and long-term | Cardiovascular- and pulmonary complications | Unknown |
| Martiny 2023 | Vasovagal reaction short-term and long-term | Cardiovascular- and pulmonary complications | Not Severe |
| Martiny 2023 | Death with follow-up time recorded | Death | Death |
| Martiny 2023 | Death without follow-up time recorded | Death | Death |
| Niv 2008 | Bleeding | Bleeding | Unknown |
| Niv 2008 | Death | Death | Death |
| Niv 2008 | Perforation | Perforation | Severe |
| Reumkens 2016 | Post-colonoscopy bleeding requiring hospitalization, need for at repeat colonoscopy, transfusion of packed red blood cells, emergency room visit | Bleeding | Unknown |
| Reumkens 2016 | “Mortality” was defined as death occurring within 3 months aft er a colonoscopy as a consequence of cardiorespiratory events, “perforation”, or “bleeding” related to the procedure. | Death | Death |
| Reumkens 2016 | Perforations requiring hospitalization or surgery | Perforation | Unknown |
| Tinmouth 2016 | Complications from tests (Perforations, bleedings and deaths) | Complications/adverse events in total | Unknown |
| Towler 1998 | Perforation | Perforation | Severe |
| Towler 1998 | Serious bleedings | Bleeding | Severe |
| Towler 1998 | Haemorrhage | Bleeding | Unknown |
| Vermeer 2017 | Major bleeding, defined as bleeding after colonoscopy requiring hospitalization, emergency room visit, need for repeat colonoscopy, transfusion of packed red blood cells or surgery | Bleeding | Very Severe |
| Vermeer 2017 | Cardiovascular events | Cardiovascular-and pulmonary complications | Unknown |
| Vermeer 2017 | Vasovagal reaction | Cardiovascular-and pulmonary complications | Not Severe |
| Vermeer 2017 | Death within 30 days after intervention | Death | Death |
| Vermeer 2017 | Discomfort | Discomfort | Not Severe |
| Vermeer 2017 | Major morbidity after colonoscopy | Other harms | Unknown |
| Vermeer 2017 | Abdominal pain | Pain | Unknown |
| Vermeer 2017 | Perforation | Perforation | Severe |

***Table 7*** *presents all extracted outcomes as defined in the original publications, the type of harm as categorized by authors AKB and FM, and the corresponding severity assessments.*

Name of data extractors: Anne Katrine Lykke Bie and Frederik Martiny
Date of data extraction (last update): December 10th 2024
