# Supplementary material for: Physical harms in colorectal cancer screening: An overview of the reporting in systematic reviews and randomised controlled trials
Source: PLoS One. 2025 Sep 11;20(9):e0331104. doi: 10.1371/journal.pone.0331104 (PMC12425235; doi:10.1371/journal.pone.0331104)
Supplement: S3 Checklist — S3 includes the PRIOR checklist, which serves as a reporting guideline for overviews of reviews of healthcare interventions, aligned with the content of the manuscript. (DOCX) [file pone.0331104.s003.docx]

**PRIOR Checklist**

**(Gates M, Gates A, Pieper D, et al. Reporting guideline for overviews of reviews of healthcare interventions:  development of the PRIOR statement. *BMJ* 2022;378:e070849. doi:10.1136/bmj-2022-070849.)**

| **Section**  Topic | **#** | **Item** | **Location  reported** |
| --- | --- | --- | --- |
| **TITLE** | | |  |
| Title | 1 | Identify the report as an overview of reviews. | 1 |
| **ABSTRACT** | | |  |
| Abstract | 2 | Provide a comprehensive and accurate summary of the purpose, methods, and results of the  overview of reviews. | 2 |
| **INTRODUCTION** | | |  |
| Rationale | 3 | Describe the rationale for conducting the overview of reviews in the context of existing knowledge. | 3 |
| Objectives | 4 | Provide an explicit statement of the objective(s) or question(s) addressed by the overview of reviews. | 5 |
| **METHODS** | | |  |
| Eligibility criteria | 5a | Specify the inclusion and exclusion criteria for the overview of reviews. If supplemental primary studies were included, this should be stated, with a rationale. | 6 + S1 Appendix C |
|  | 5b | Specify the definition of ‘systematic review’ as used in the inclusion criteria for the overview of reviews. | 5 |
| Information  sources | 6 | Specify all databases, registers, websites, organizations, reference lists, and other sources searched  or consulted to identify systematic reviews and supplemental primary studies (if included).  Specify the date when each source was last searched or consulted. | 5 |
| Search strategy | 7 | Present the full search strategies for all databases, registers and websites, such that they could be reproduced. Describe any search filters and limits applied. | 4+S1 Appendix B |
| Selection process | 8a | Describe the methods used to decide whether a systematic review or supplemental primary study  (if included) met the inclusion criteria of the overview of reviews. | 6+S1 Appendix C |
|  | 8b | Describe how overlap in the populations, interventions, comparators, and/or outcomes of  systematic reviews was identified and managed during study selection. | 6 |
| Data collection  process | 9a | Describe the methods used to collect data from reports. | 6 |
|  | 9b | If applicable, describe the methods used to identify and manage primary study overlap at the level  of the comparison and outcome during data collection. For each outcome, specify the method used  to illustrate and/or quantify the degree of primary study overlap across systematic reviews. | 6 |
|  | 9c | If applicable, specify the methods used to manage discrepant data across systematic reviews  during data collection. | Not applicable |
| Data items | 10 | List and define all variables and outcomes for which data were sought. Describe any assumptions  made and/or measures taken to identify and clarify missing or unclear information. | 6+ S1 Appendix D |
| Risk of bias  assessment | 11a | Describe the methods used to *assess* risk of bias or methodological quality of the included  systematic reviews. | 7 |
|  | 11b | Describe the methods used to *collect* data on (from the systematic reviews) and/or *assess* the risk  of bias of the primary studies included in the systematic reviews. Provide a justification for  instances where flawed, incomplete, or missing assessments are identified but not re-assessed. | 7 |
|  | 11c | Describe the methods used to *assess* the risk of bias of supplemental primary studies (if included). | 7 |
| Synthesis  methods | 12a | Describe the methods used to summarize or synthesize results and provide a rationale for the  choice(s). | 6 |
|  | 12b | Describe any methods used to explore possible causes of heterogeneity among results. | Not applicable |
|  | 12c | Describe any sensitivity analyses conducted to assess the robustness of the synthesized results. | Not applicable |
| Reporting bias  assessment | 13 | Describe the methods used to *collect* data on (from the systematic reviews) and/or *assess* the risk  of bias due to missing results in a summary or synthesis (arising from reporting biases at the levels  of the systematic reviews, primary studies, and supplemental primary studies, if included). | 5-7 |
| Certainty  assessment | 14 | Describe the methods used to *collect* data on (from the systematic reviews) and/or *assess* certainty  (or confidence) in the body of evidence for an outcome. | 6 |
| **RESULTS** | | |  |
| Systematic review  and supplemental  primary study  selection | 15a | Describe the results of the search and selection process, including the number of records screened,  assessed for eligibility, and included in the overview of reviews, ideally with a flow diagram. | 9-14 |
|  | 15b | Provide a list of studies that might appear to meet the inclusion criteria, but were excluded, with  the main reason for exclusion. | S1 Appendix F(a) and F(b) |

| **Section**  Topic | **#** | **Item** | **Location  reported** |
| --- | --- | --- | --- |
| Characteristics of  systematic  reviews and  supplemental  primary studies | 16 | Cite each included systematic review and supplemental primary study (if included) and present its  characteristics. | 11-14 (table 1b)  RCTs are cited at page 9-10 (table 1a) |
| Primary study  overlap | 17 | Describe the extent of primary study overlap across the included systematic reviews. | 6 |
| Risk of bias in  systematic  reviews, primary  studies, and  supplemental  primary studies | 18a | Present assessments of risk of bias or methodological quality for each included systematic review. | NA (p. 7) |
|  | 18b | Present assessments (*collected* from systematic reviews or *assessed* anew) of the risk of bias of  the primary studies included in the systematic reviews. | NA (p. 7) |
|  | 18c | Present assessments of the risk of bias of supplemental primary studies (if included). | NA (p. 7) |
| Summary or  synthesis of  results | 19a | For all outcomes, summarize the evidence from the systematic reviews and supplemental primary studies (if included). If meta-analyses were done, present for each the summary estimate and its precision and measures of statistical heterogeneity. If comparing groups, describe the direction of the effect. | 15-24 |
|  | 19b | If meta-analyses were done, present results of all investigations of possible causes of  heterogeneity. | NA |
|  | 19c | If meta-analyses were done, present results of all sensitivity analyses conducted to assess the  robustness of synthesized results. | NA |
| Reporting biases | 20 | Present assessments (*collected* from systematic reviews and/or *assessed* anew) of the risk of bias  due to missing primary studies, analyses, or results in a summary or synthesis (arising from  reporting biases at the levels of the systematic reviews, primary studies, and supplemental primary  studies, if included) for each summary or synthesis assessed. | 27-28 |
| Certainty of  evidence | 21 | Present assessments (*collected* or *assessed* anew) of certainty (or confidence) in the body of  evidence for each outcome. | NA (p. 6) |
| **DISCUSSION** | | |  |
| Discussion | 22a | Summarize the main findings, including any discrepancies in findings across the included  systematic reviews and supplemental primary studies (if included). | 24-25 |
|  | 22b | Provide a general interpretation of the results in the context of other evidence. | 25-27 |
|  | 22c | Discuss any limitations of the evidence from systematic reviews, their primary studies, and  supplemental primary studies (if included) included in the overview of reviews. Discuss any  limitations of the overview of reviews methods used. | 25-26 |
|  | 22d | Discuss implications for practice, policy, and future research (both systematic reviews and  primary research). Consider the relevance of the findings to the end users of the overview of  reviews, e.g., healthcare providers, policymakers, patients, among others. | 28-30 |
| **OTHER INFORMATION** | | |  |
| Registration and  protocol | 23a | Provide registration information for the overview of reviews, including register name and  registration number, or state that the overview of reviews was not registered. | 5 |
|  | 23b | Indicate where the overview of reviews protocol can be accessed, or state that a protocol was not  prepared. | 5 |
|  | 23c | Describe and explain any amendments to information provided at registration or in the protocol.  Indicate the stage of the overview of reviews at which amendments were made. | 5 |
| Support | 24 | Describe sources of financial or non-financial support for the overview of reviews, and the role of  the funders or sponsors in the overview of reviews. | 31 |
| Competing  interests | 25 | Declare any competing interests of the overview of reviews' authors. | 31 |
| Author  information | 26a | Provide contact information for the corresponding author. | 1 |
|  | 26b | Describe the contributions of individual authors and identify the guarantor of the overview of  reviews. | 1 |
| Availability of  data and other  materials | 27 | Report which of the following are available, where they can be found, and under which conditions  they may be accessed: template data collection forms; data collected from included systematic  reviews and supplemental primary studies; analytic code; any other materials used in the overview  of reviews. | All data can be found in the included appendices  In S1 and S2 |
