# Supplementary material for: Physical harms in colorectal cancer screening: An overview of the reporting in systematic reviews and randomised controlled trials
Source: PLoS One. 2025 Sep 11;20(9):e0331104. doi: 10.1371/journal.pone.0331104 (PMC12425235; doi:10.1371/journal.pone.0331104)
Supplement: Attachment — Submitted filename: Response to reviewers.docx [file pone.0331104.s006.docx]

## Dear EDITOR

Thank you for considering our responses to peer review and our revised manuscript for publication in PLOS One. Below, we respond to your comments about the journal requirement and to the comments from the peer reviewers. We have responded to all reviewer feedback, in bold font below, and revised the manuscript accordingly where relevant.

Response to reviewers

6. Review Comments to the Author

Reviewer #1: I had just two comments on the original version of the article.
The authors addressed one of them, regarding the formatting of some tables.
The other comment was not fully addressed (the incidence of the unreported harms) but the authors provided a sensible reason for not doing so.
I think the article is ready for publication.

**Thank you so much for your valued comments.**

Reviewer #2: Thank you for the revised version of the manuscript. The authors have addressed the previous comments in a satisfactory manner. Overall, the manuscript has improved in both content and presentation. I believe it is suitable for publication after minor revision.
In line 10, the quotation mark (") at the beginning of the paragraph appears to be unnecessary or mistakenly placed, as it is not closed later in the text. The authors are kindly requested to check the manuscript for clarity, consistency, and minor formatting issues.
Implications for screening participants
10 "We believe it is important…..

**We thank the reviewer for their careful and attentive reading of the manuscript. The typographical error has been corrected as suggested. Additionally, we have reviewed the manuscript and the additional files for any remaining typos or grammatical errors. All changes are marked in tracked changes in the uploaded manuscript file.**
